# Intestinal Protein Characterisation of SARS-CoV-2 Entry Molecules ACE2 and TMPRSS2 in Inflammatory Bowel Disease (IBD) and Fatal COVID-19 Infection

**DOI:** 10.1007/s10753-021-01567-z

**Published:** 2021-10-25

**Authors:** Milly J. McAllister, Kathryn Kirkwood, Shaun C. Chuah, Emily J. Thompson, Jennifer A. Cartwright, Clark D. Russell, David A. Dorward, Christopher D. Lucas, Gwo-tzer Ho

**Affiliations:** 1 Centre for Inflammation Research, Queens Medical Research Institute, University of Edinburgh, 47 Little France Crescent, Edinburgh, EH16 4TJ UK; 2 Gastroenterology and Pathology Department, Western General Hospital, Crewe Road South, Edinburgh, EH4 2XU UK; 3 Regional Infectious Diseases Unit, Western General Hospital, Crewe Road South, Edinburgh, EH4 2XU UK; 4 Department of Pathology, Royal Infirmary of Edinburgh, 51 Little France Crescent, Edinburgh, EH16 4SA UK; 5 Department of Respiratory Medicine, Royal Infirmary of Edinburgh, 51 Little France Crescent, Edinburgh, EH16 4SA UK; 6 Edinburgh IBD Science Unit, Centre for Inflammation Research, University of Edinburgh, Edinburgh, Scotland, UK

**Keywords:** COVID-19, SARS-CoV-2, IBD, UC, Crohn’s disease, inflammation, gut biology

## Abstract

The coronavirus SARS-CoV-2 contributes to morbidity and mortality mainly as a result of immune-pathology in the lungs. Recent data has shown multi-system involvement with widespread viral tropism. Here we present a detailed intestinal protein characterisation of SARS-Cov-2 entry molecules ACE2 and TMPRSS2 in patients with inflammatory bowel disease ([IBD]; ulcerative colitis [UC] and Crohn’s disease [CD]) with age- and sex-matched non-IBD controls, and in those with fatal COVID-19 infection. In our dataset, ACE2 and TMPRSS2 displayed a membrane enterocyte staining in the ileum (due to presence of brush border/microvilli) in contrast to a cytoplasmic pattern in the colon. We also showed a high ACE2/low TMPRSS2 expression pattern in the ileum with a reverse trend in the colon. In UC, colonic ACE2 and TMPRSS2 are cytoplasmic in nature, with significantly higher ACE2 staining intensity compared to non-IBD controls. In inflamed and unaffected IBD mucosa, ileal and colonic enterocyte ACE2 and TMPRSS2 expressions are not modified in the histologic presence of inflammation. We observed immune cells within the lamina propria that expressed ACE2 and TMPRSS2, at higher frequencies in IBD when compared to non-IBD controls. These were identified as plasma cells with multiple myeloma oncogene 1/interferon regulatory factor 4 (MUM1/IRF4) expression. We further analysed the gut histology of six fatal COVID-19 cases, with no difference in colonic and ileal ACE2/TMRPSS2 staining (compared to non-IBD controls) and identified ACE2 + lamina propria plasma cells. Of interest, in this COVID-19 cohort, there was no histologic evidence gut inflammation despite known evidence of viral tropism within the enterocytes. Our data provides evidence for tissue expression of entry molecules ACE2 and TMPRSS2 including a close apposition to plasma cells — both pointing towards a role of the gut in the antecedent immune response to SARS-CoV-2 infection.

**Supplementary Information:**

The online version contains supplementary material available at 10.1007/s10753-021-01567-z.

The pathogenic mechanisms of severe acute respiratory syndrome coronavirus 2 (SARS-CoV-2) in mediating the clinical syndrome of COVID-19 are increasingly better understood. The SARS-Cov-2 virus gains entry to host cells upon binding of its spike (S) proteins to the angiotensin I converting enzyme 2 (ACE2) receptor where the transmembrane protease (TMPRSS2) primes the S proteins to facilitate this process [1]. Although mortality and morbidity associated with COVID-19 are driven largely by immunopathology in the lung, viral protein and RNA are detected throughout the body [2]. Across human tissue and organs, ACE2 and TMPRSS2 are highly expressed within the gastrointestinal tract where transcriptomics analysis shows highest gene expressions in the ileal and colonic enterocytes [3]. Diarrhoea is the most common GI symptoms and there is considerable debate whether this is linked to more severe COVID-19 (reviewed in [4]). Pertinently, two recent questions arise on the importance of the gastrointestinal tract and its role in faecal-oral transmission and as a site of host immune response to SARS-CoV-2. Of immediate relevance is the potential importance of local tissue factors such as the presence of chronic inflammation and in common chronic immune-mediated gastrointestinal conditions such as inflammatory bowel diseases (IBD) comprising of ulcerative colitis (UC) and Crohn’s disease (CD) that affect more than 10 million people globally. In this context, we characterised the protein expression of ACE2 and TMPRSS2 in individuals with UC and CD (each with inflamed and unaffected areas intestinal mucosa from surgically resections); and also post-mortem intestinal tissue in those with fatal COVID-19 (*n* = 11, 10 and 6 respectively; East of Scotland Ethical Review No 20/ES/0061 and 16/ES/0084 for IBD and COVID-19-ICECAP post-mortem studies respectively).

In our study, intestinal protein ACE2 and TMPRSS2 show a membrane and cytoplasmic staining pattern in the ileum (due to presence of brush border/microvilli) and colon respectively (Fig. 1A). Overall, ACE2 expression is significantly higher in the ileum compared to colon, while TMPRSS2 is lower. We performed in silico analysis of our colonic gene microarray dataset (99 CD, 129 UC and 50 non-IBD controls; available at Gene Expression Omnibus (http://www.ncbi.nlm.nih.gov/geo/ [accessed September 2020] accession: GSE11223 and GSE20881) [5] to demonstrate *ACE2* gene expression in whole gut mucosal biopsies is significantly higher in the ileum compared with colon, with the reverse pattern seen for *TMPRSS2* (Fig. 1B). Further sub-group analysis showed this pattern of *ACE2*_*high*_*/TMPRSS2*_*low*_ ratio is noted in the overall IBD and CD groups in biopsies from the ileum when compared to non-IBD controls (Supp Fig. 1), consistent with recent studies in different IBD cohorts [6, 7]. We showed that ACE2_high_/TMPRSS2_low_ in ileum vs. colon is observed in membrane but not cytoplasmic protein expression (Fig. 1B). In UC, cytoplasmic ACE2 is significantly higher compared to non-IBD controls (Fig. 1D), whereas in CD, this pattern is observed only in membrane ACE. ACE2 and TMPRSS2 are not significantly different in inflamed and unaffected parts of the colon and ileum suggesting that active IBD does not modify the expressions of these proteins (Fig. 1D). Our gene microarray dataset (GSE11223 and GSE20881) also showed no overall differences in ileo-colonic *ACE2* and *TMPRSS2* expression in inflamed and uninflamed mucosal biopsies in UC and CD in support of this (Fig. 1E).

**Fig. 1:**
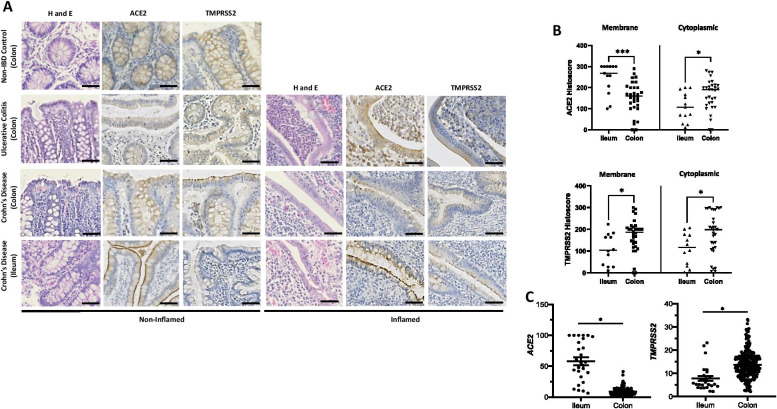

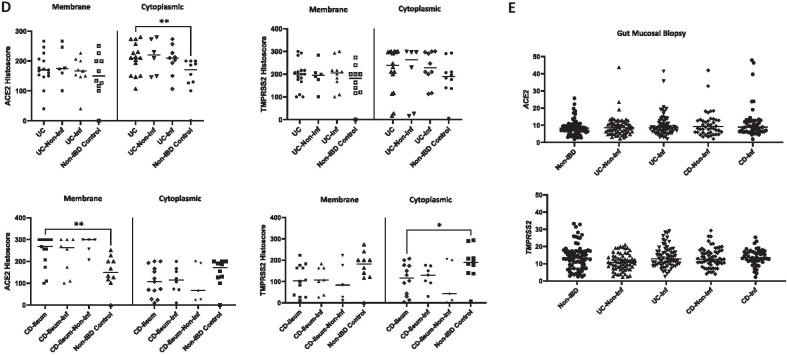
ACE2 and TMPRSS2 protein and gene expressions in ulcerative colitis (UC), and Crohn’s disease (CD) patients and non-IBD controls. **A** H&E and immunohistochemistry of ACE2 and TMPRSS2 protein expression non-IBD (*n* = 10), UC (all colon) and CD (ileum and colon) (*n* = 11) patients. Representative images of non-inflamed and inflamed colonic and ileal mucosal sections. **B** ACE2 and TMPRSS2 protein weighted histoscore expressions within membrane and cytoplasm of intestinal enterocyte in ileum and colon (total *n* = 13 mucosal CD sections for ileum; total *n* = 32 mucosal colonic — 22 UC and 10 non-IBD colon); ***p = 0.005 and *p < 0.05. **C** *ACE2* and *TMPRSS2* gene expressions in ileum and colon whole mucosal gut pinch biopsies†; 27 ileal (*n* = 15, 5 and 7 CD, UC and non-IBD biopsies respectively) and 273 colonic (*n* = 83, 129 and 66 CD, UC and non-IBD biopsies respectively); *p < 0.0001. **D** Membrane and cytoplasmic ACE2 and TMPRSS2 in UC and CD compared to non-IBD controls; **p < 0.01, *p < 0.05. **E** Inflamed and non-inflamed gut *ACE2* and *TMPRSS2* gene expressions in UC, CD and non-IBD† (non-IBD, UC-Non-Inf, UC-Inf, CD-Non-Inf and CD-Inf — *n* = 73, 62, 67, 48 and 50 mucosal biopsies respectively). †Data accessed from microarray dataset http://www.ncbi.nlm.nih.gov/geo/ accession: GSE11223 and GSE20881. Only significant p-values presented. Non-Inf, non-inflamed mucosa; Inf, inflamed mucosa.

Of interest, we observed immune cells within the lamina propria in the ileum and colon that expressed ACE2 and TMPRSS2, more commonly found in UC and CD compared with non-IBD controls (Fig. 2A and B). On histopathological review (GI-pathologist, KK), these were identified as plasma cells together with the finding of protein co-localisation with multiple myeloma oncogene 1/interferon regulatory factor 4 (MUM1/IRF4) expression, a commonly used plasma cell marker (Fig. 2C and D). We further analysed the gut histology of six fatal COVID-19 cases (using tissue generated from the ICECAP post-mortem study) and identified MUM1 + plasma cells co-localised with ACE2 within the lamina propria in the ileum and colon from our COVID-19 cohort (Fig. 2E). In this cohort, there was no gut inflammation despite known evidence of Sars-CoV-2 viral tropism within the enterocytes [2]. In addition, ACE2 and TMPRSS2 enterocyte expressions are not different in COVID-19 compared to non-IBD controls (Fig. 2G).

**Fig. 2:**
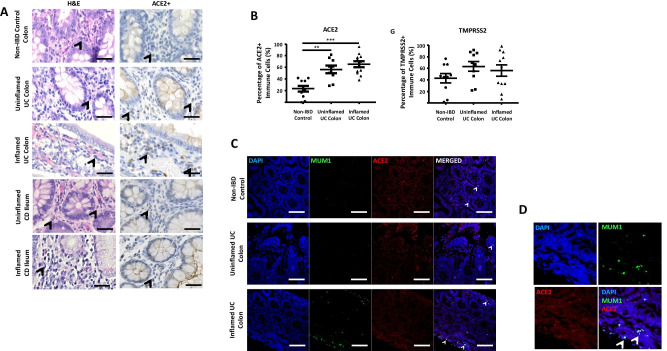

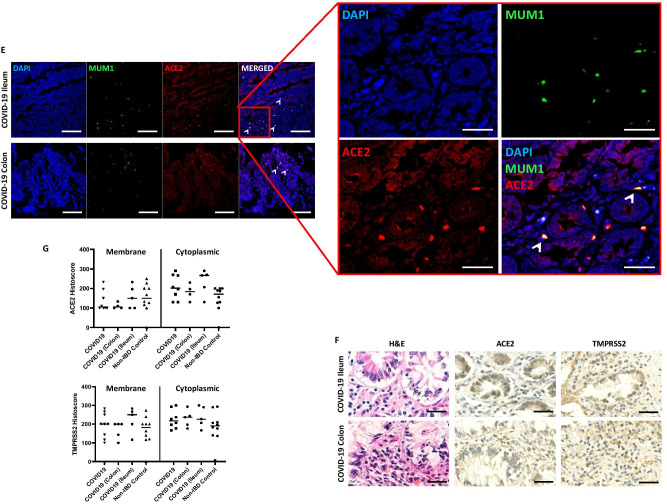
**A** H&E and immunohistochemistry of ACE2 in non-IBD (*n* = 10), UC (all colon) and CD (ileum and colon) (*n* = 11) patients. Representative images of non-inflamed and inflamed colonic and ileal mucosal sections. Arrowhead indicating plasma cells. **B** ACE2 and TMPRSS2 positive cells (%) within lamina propria in inflamed and uninflamed colon of UC vs. non-IBD controls (**p < 0.001 and *** *p* < 0.0001). **C**, **D** Immunofluorescence co-staining of ACE2 (Red), MUM1 (Green), and DAPI (Blue) as nuclear stain within the lamina propria of in inflamed and uninflamed colon of UC vs. non-IBD controls. **E** Immunofluorescence co-staining of ACE2 (Red), MUM1 (Green) and DAPI (Blue) as nuclear stain within the lamina propria of expression in fatal COVID-19 (SARS-CoV-2 PCR positive) ileum and colon sections; and non-IBD controls. **F** H&E and immunohistochemistry of ACE2 and TMPRSS2 protein expression in fatal COVID-19 (COVID-19 colon and ileum — *n* = 4 and 5 mucosal sections respectively). **G** ACE2 and TMPRSS2 protein weighted histoscore expressions within membrane and cytoplasm of intestinal enterocyte (COVID-19 colon and ileum — *n* = 4 and 5 mucosal sections respectively and 9 non-IBD colon).

Reassuringly to date, clinical databases such as SECURE-IBD [8] have not shown an increase in susceptibility or severity of COVID-19 in patients with IBD. Our report which focused on both protein and gene expression data showed that mucosal inflammation was not associated with changes in the ACE2 and TMPRSS2. Published gene expression in different IBD datasets has shown either no change or a trend towards an increase in colonic ACE2 expression in IBD with some correlation with inflammation and region of large bowel studied [6, 7, 9–11]. In an early human tissue characterisation study, ACE2 protein was found to be highly expressed within the brush border/apical membrane of the small intestine, and not seen in the colon [12]. Recent human intestinal protein studies on ACE2 and TMPRSS2 (also in small IBD cohorts like our patient group) [7, 10, 13] confirm the former but showed patchy colonic expression for ACE2. Of interest, we identified high cytoplasmic protein ACE2 and TMPRSS2 in the colonic epithelium in UC. The biological significance of cytoplasmic localisation of ACE is unclear and more work is necessary. ACE2 may have a wider role in modulating gut homeostasis, microbiota and inflammatory response (as recently reviewed [14]).

Our finding of plasma cells expressing ACE2 in inflamed IBD gut is noteworthy. Recent studies point to the importance of the humoral immune response, particularly IgA neutralising antibodies to SARS-CoV-2, which is more potent, occurs rapidly and remains more persistent. Gaebler et al. show that SARS-CoV-2 antigen persistence in the gut may be the key factor that drives antibody evolution and potency [15]. COVID-19 patients have been shown to develop metabolically hyperactive plasmablasts during inflammatory states [16]. IgA antibodies dominated the early SARS-CoV-2–specific antibody response compared with IgG and IgM and were associated with expansion of IgA plasmablasts with mucosal homing characteristics [17]. Although our primary data does not show gut inflammation as a specific factor for SARS-CoV-2 entry mechanism, the high expression of ACE2 and TMPRSS2 along with the apposition of plasma cells in the gut lamina propria suggests that the gastrointestinal tract may play a role in SARS-CoV-2 entry and the antecedent host humoral immune response in COVID-19.

## Supplementary Information

Below is the link to the electronic supplementary material.

Supplementary file1 (DOCX 42 KB)

Supplementary file2 ACE2 and TMPRSS2 gene expression on ileum and colon mucosal biopsies. Data accessed from microarray dataset http://www.ncbi.nlm.nih.gov/geo/ accession: GSE11223 and GSE20881. p-values presented. (PPTX 426 KB)

## Data Availability

Full data presented in this data is available on request to corresponding author.
