# Supplementary material for: Intestinal Protein Characterisation of SARS-CoV-2 Entry Molecules ACE2 and TMPRSS2 in Inflammatory Bowel Disease (IBD) and Fatal COVID-19 Infection
Source: Inflammation. 2021 Oct 25;45(2):567–72. doi: 10.1007/s10753-021-01567-z (PMC8545358; doi:10.1007/s10753-021-01567-z)
Supplement: Supplementary file 1 — Supplementary file1 (DOCX 42 KB) [file 10753_2021_1567_MOESM1_ESM.docx]

**Supplementary information**

**Methodology**

**Patient cohorts**

Intestinal resection specimens from 21 patients with inflammatory bowel disease (Crohn’s disease *n*=11 [ileum *n*=8, colon *n*=3], Ulcerative Colitis n=10 [colon n=10]) and 10 non-IBD control patients (colon resection for colorectal cancer) were studied. All surgical resections were carried out between 1^st^ Jan 2016 and 31^st^ December 2020 in the Western General Hospital, Edinburgh (East of Scotland Research Ethical Approval No 20/ES/0061). The groups were matched for age- and gender; mean age 66, 64 and 68 years old for UC, CD and non-IBD controls at time of resection respectively. Histological analysis of inflamed vs. non-inflamed areas in IBD resection specimens were carried out by GI pathologist (KK).

Additionally, enteric tissue samples were obtained from post-mortem examinations conducted on six patients with pre-mortem PCR-confirmed SARS-CoV-2 infection a median of 19.6 hours (IQR 13.6-47.2) after death, as previously reported^2^ (East of Scotland Research Ethical Approval No 16/ES/0084). Formalin-fixed paraffin-embedded (FFPE) tissue blocks were prepared following standard processes. Patients with Covid-19 were all male and had a mean age of 71 years. None of the patients had pre-existing intestinal disease. All patients had hypoxic respiratory failure with radiological evidence of viral pneumonitis. SARS-CoV-2 was present in colon tissue from all six patients (detected using multiplex PCR using ARTIC Network protocol, confirmed by sequencing, as described with subgenomic messenger RNA detected in 5/6, indicative of viral RNA synthesis.

**Immunohistochemistry**

Formalin-fixed paraffin-embedded (FFPE) tissues biopsies were processed into 4µm sections and stained for haematoxylin and eosin. Sections were de-paraffinised in xylene, rehydrated through graded alcohol, and heated for 5 minutes under pressure for antigen retrieval in Citrate buffer pH6 (2mM Sodium Citrate and 8mM Citric Acid). Endogenous peroxidase activity was blocked using 3% H_2_O_2_ for 30 minutes and non-specific background staining was blocked for 30 minutes in 10% horse serum in Tris-buffered saline (TBS). Primary antibodies for ACE2 (Cat Ab15348; Abcam) and TMPRSS2 (Cat Ab92323; Abcam) were incubated overnight at 4^o^C diluted at 1:1000. Sections were incubated in ImmPRESS detection kit (Vector Laboratories, UK) and visualised using 3,3-diaminobenzidine tetrahydrochloride (DAB, Dako, UK). Samples were then counterstained, dehydrated, and mounted. Antibody specificity for ACE2 and TMPRSS2 was confirmed using an IgG isotype control (Cat Ab172730; Abcam). Slides were scanned using Zeiss AxioScanner (Zeiss, Germany) and analysed using Carl Zeiss Zen 2.6 Blue edition programme (Zeiss, Germany). Staining intensity for ACE2 and TMPRSS2 was performed using a weighted histo-score method for cytoplasmic and membrane expression in the epithelia and lamina propria. In brief, the score was calculated by sum of (1 X % cells staining weakly positive) + (2 X % cells staining moderately positive) + (3 X % cells staining strongly positive) with a maximum of 300 (100% strongly stained) and a minimum of 0 (100% with no staining). The percentage of ACE2 or TMPRSS2 positive immune cells within the lamina propria was calculated by counting the percentage of positive cells within three representative 0.6mm x 0.6mm areas. ACE2 and TMPRSS2 staining intensity and positive immune cell counts were scored by two independent observers.

**Immunofluorescence**

Tissue sections were de-paraffinised and rehydrated before undergoing antigen retrieval in Citrate Buffer pH6. Samples were blocked for 1 hour using 2% fetal calf serum and incubated in primary antibodies ACE2 (1:1000, Cat Ab15348; Abcam) and Anti-MUM1 (1:100, Cat Ab247079; Abcam) overnight at 4^o^C and for 1 hour at room temperature receptively. Sections were incubated in Alexa Fluor® 488 (Cat Ab150077; Abcam) and Alexa Fluor® 555 (Cat Ab150078; Abcam) receptively and visualised using Zeiss LSM 780 Confocal followed by analysis on Carl Zeiss ZEN 2 blue edition software (Zeiss, Germany). Staining was categorised based on absence and/ or presence of ACE2 and/ or MUM1, analysed in three representative 0.6mm X 0.6mm areas within the lamina propria, and scored by two independent observers.

**Gene** **microarray**

Gene expression for *ACE2* and *TMPRSS2* were analysed from whole gut endoscopic pinch biopsies taken from 99 Crohn’s Disease, 129 Ulcerative Colitis and 50 non-IBD control patients (Gene microarray data was available for total of 300 mucosal biopsies). Full details of tissue acquisition and processing are previously detailed^5^. The whole data set is available at Gene Expression Omnibus (<http://www.ncbi.nlm.nih.gov/geo/> (accessed September 2020) accession: GSE11223 and GSE20881.

**Statistics**

Statistical analysis was performed using SPSS Version 25.0 (IBM, USA) and GraphPad Prism Version 9 (GraphPad Software, USA) for Mann-Whitney U-Test. Interclass correlation coefficients were employed to confirm two independent scores of tissues staining intensity.
