# Supplementary figures and images for: Intestinal Protein Characterisation of SARS-CoV-2 Entry Molecules ACE2 and TMPRSS2 in Inflammatory Bowel Disease (IBD) and Fatal COVID-19 Infection
Source: Inflammation. 2021 Oct 25;45(2):567–72. doi: 10.1007/s10753-021-01567-z (PMC8545358; doi:10.1007/s10753-021-01567-z)

## Slide 1
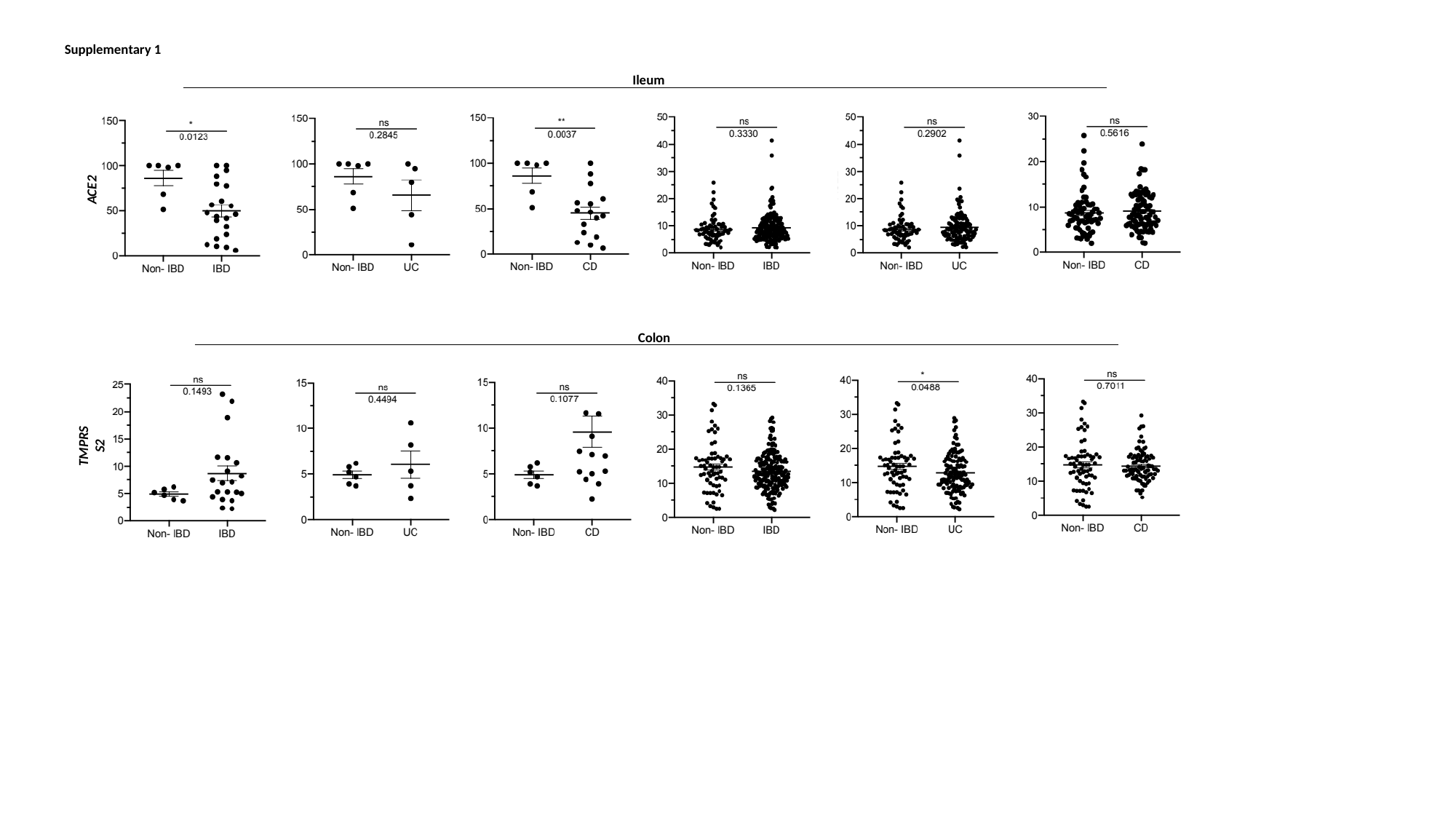

Supplementary 1
Ileum
ACE2
Colon
TMPRSS2

Supplement: Supplementary file 2 — Supplementary file2 ACE2 and TMPRSS2 gene expression on ileum and colon mucosal biopsies. Data accessed from microarray dataset http://www.ncbi.nlm.nih.gov/geo/ accession: GSE11223 and GSE20881. p-values presented. (PPTX 426 KB) [file 10753_2021_1567_MOESM2_ESM.pptx]
